# Conformational states during vinculin unlocking differentially regulate focal adhesion properties

**DOI:** 10.1038/s41598-018-21006-8

**Published:** 2018-02-09

**Authors:** Dror S. Chorev, Tova Volberg, Ariel Livne, Miriam Eisenstein, Bruno Martins, Zvi Kam, Brigitte M. Jockusch, Ohad Medalia, Michal Sharon, Benny Geiger

**Affiliations:** 1 Department of Molecular Cell Biology, Weizmann Institute of Science, Rehovot, 7610001 Israel; 2 Department of Biomolecular Sciences, Weizmann Institute of Science, Rehovot, 7610001 Israel; 3 Chemical Research Support, Weizmann Institute of Science, Rehovot, 7610001 Israel; 4 Department of Biochemistry, University of Zurich, Winterthurerstrasse 190, Zurich, 8057 Switzerland; 5 Department of Cell Biology, Zoological Institute, Technical University of Braunschweig, D-38092 Braunschweig, Germany; 6 Department of Life Sciences and the National Institute for Biotechnology in the Negev, Ben-Gurion University, Beer-Sheva, 84105 Israel

## Abstract

Focal adhesions (FAs) are multi-protein complexes that connect the actin cytoskeleton to the extracellular matrix, via integrin receptors. The growth, stability and adhesive functionality of these structures are tightly regulated by mechanical stress, yet, despite the extensive characterization of the integrin adhesome, the detailed molecular mechanisms underlying FA mechanosensitivity are still unclear. Besides talin, another key candidate for regulating FA-associated mechanosensing, is vinculin, a prominent FA component, which possesses either closed (“auto-inhibited”) or open (“active”) conformation. A direct experimental demonstration, however, of the conformational transition between the two states is still absent. In this study, we combined multiple structural and biological approaches to probe the transition from the auto-inhibited to the active conformation, and determine its effects on FA structure and dynamics. We further show that the transition from a closed to an open conformation requires two sequential steps that can differentially regulate FA growth and stability.

## Introduction

Focal adhesions (FA) are trans-membrane multi-protein complexes that anchor the actin cytoskeleton to the underlying extracellular matrix (ECM) via integrin adhesion receptors^1,2^. FA formation is initiated by activated integrin heterodimers, which bind to the ECM through their extracellular domains, and are linked to the intracellular actin cytoskeleton via a multitude of scaffolding and signaling proteins, collectively known as the integrin adhesome^3–8^. The assembly and maturation of FAs involves multiple molecular interactions, governed by internal and external forces^9–11^.

After an initial engagement of integrin dimers with the ECM at nascent adhesions, a dimer of the adaptor protein talin, binds to the cytoplasmic tails of integrins, driving the force-dependent recruitment of multiple adhesome components^12^. This is done by interaction of talin with the membrane and the actin cytoskeleton that leads to physical stress, which induces the exposure of several vinculin binding sites on talin’s rod domain. Similarly, local forces, acting on the nascent adhesions are believed to induce a conformational change within the vinculin molecule, causing its transition from an auto inhibited, closed conformation to an active, open one^13,14^, enabling it to bind to talin and further recruit additional adhesome components. It was further shown that similar vinculin conformational activation process occurs within cadherin-mediated cell-cell adherens junctions, where the “open” vinculin binds to α-catenin^15–18^. This process is followed by recruitment of additional adhesome components including mechanosensitive proteins, like focal adhesion kinase (FAK) and p130Cas which participate in FA regulation, following the exposure of their kinase domain, and sequestered phosphorylation sites, respectively^19,20^.

**Figure 1:**
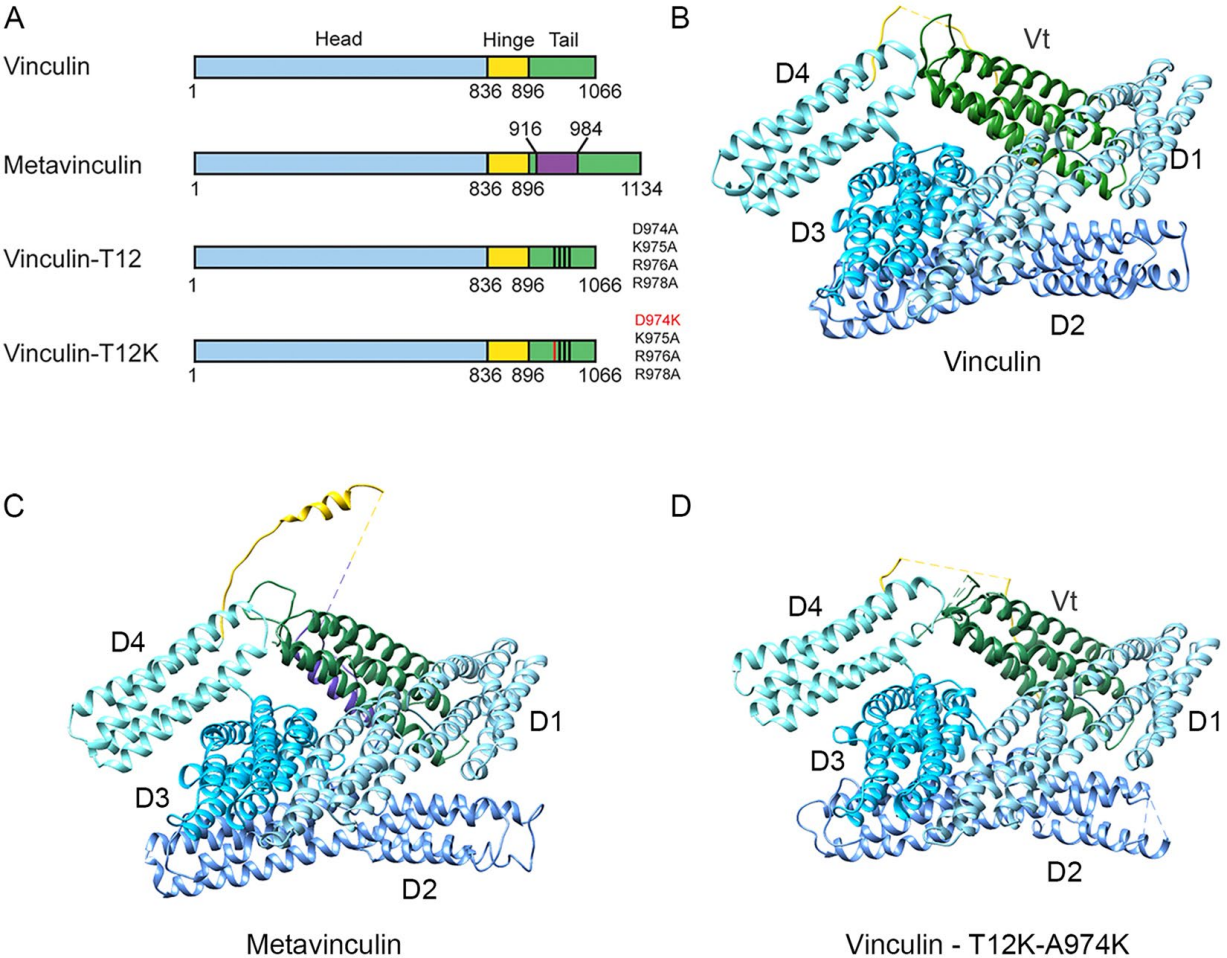
The three dimensional structures of vinculin variants. (**A**) Schematic illustration of the different vinculin variants, depicting the head (blue), hinge (yellow) and tail (green) domains and the sequence variation between the forms. The 68 amino acid insert of metavinculin is labeled in purple. (**B**–**D**) Ribbon depictions of the X-ray structures of vinculin (PDB code 1TR2), metavinculin^30^ and vinculin-T12-A974K (solved in this study) showing the very similar overall fold. The head, hinge, metavinculin insert and tail demains are colored as in 1 A. Missing segments, which were not resolved in the electron density map, are shown as dashed lines.

In this study we focused on the mechanism underlying vinculin activation. Vinculin is composed of a N-terminal globular head domain, which consists of 4 α-helical bundles (D1-D4), that are connected to a C-terminal tail via a flexible hinge (Fig. 1A)^21,22^. The crystal structure of vinculin has revealed that the head domain interacts with the tail, forming the stable closed, “auto-inhibited” conformation, which needs to open-up in order to interact with talin^14,22^. The exact mechanism whereby vinculin is activated is unclear^23^, yet, the biochemical evidence that have accumulated so far are in line with the view that interruption of intramolecular contacts between the tail and both D1 and D4 head domains is needed for vinculin activation^14^.

To investigate the molecular mechanics that mediates vinculin’s transition from the closed to the open state we have compared the energy levels required for opening wild-type (WT) vinculin, to those required for opening a mutant vinculin molecule, termed vinculin-T12, in which the head-binding surface on the vinculin tail was mutated^14^ (Fig. 1), thereby weakening the head-to-tail interaction affinity by a 100-fold^14^. Assays inspecting the kinetics of this mutant have shown that the lifetime of vinculin-T12 in FAs is three times longer than that of the WT molecule, suggesting that vinculin-T12 displays an open conformation, exposing binding sites to diverse associated proteins. Further investigation revealed that the presence of vinculin-T12 leads to an extended lifespan of multiple adhesion components in FAs^13,24^, as well as enhanced cell spreading, surface traction and adhesion strength^25^.

In addition to comparing the energy required to open vinculin to that of vinculin-T12, we have created a novel variant of T12 termed T12-A974K, which contains an additional positive charge that might further destabilize head-to-tail interactions, leading to facilitated activation. We also explored the contribution of the hinge region that interconnects the head to the tail, to vinculin activation, by studying the splice isoform metavinculin (mVin). This vinculin isoform contains an extra acidic stretch of 68-amino acids, placed between the tail and the flexible hinge^26–28^ (Fig. 1A). mVin is present only in smooth, cardiac and skeletal muscle cells, where the cytoskeletal force regime might differ from that of non-muscle cells. Previous work suggested that the extra segment of mVin interferes with the auto-inhibitory head-tail interaction and the binding to other classical partners, as well as the ability to specifically bind lipids^29^.

In this report, we combine crystallographic studies, native mass-spectrometry (MS), ion-mobility MS (IM-MS) measurements, molecular modeling and cell biological characterization, aiming to understand the mechanism of vinculin activation. We demonstrate that all three variants (T12, T12-A974K and mVin) exist in a closed conformation, similar to that of native vinculin. However, unlike vinculin, T12 and T12-A974K that reside predominantly in closed conformation, mVin coexists in a diverse ensemble of states, ranging from the closed state to a range of extended conformations. In addition, collision-induced unfolding experiments coupled with IM-MS measurements show that the opening of all vinculin forms is governed by a two-step unlocking of the head to tail interaction, revealing a distinct “semi-open” conformational state of the molecule. The functional implications of these results are further highlighted by cellular analysis that relates specific steps in the unfolding process to the formation, stability and mechanosensitivity of focal adhesions.

## Results

### A closed head-to-tail conformation is revealed in the crystal structures of vinculin, metavinculin and vinculin-T12-A974K

**Figure 2:**
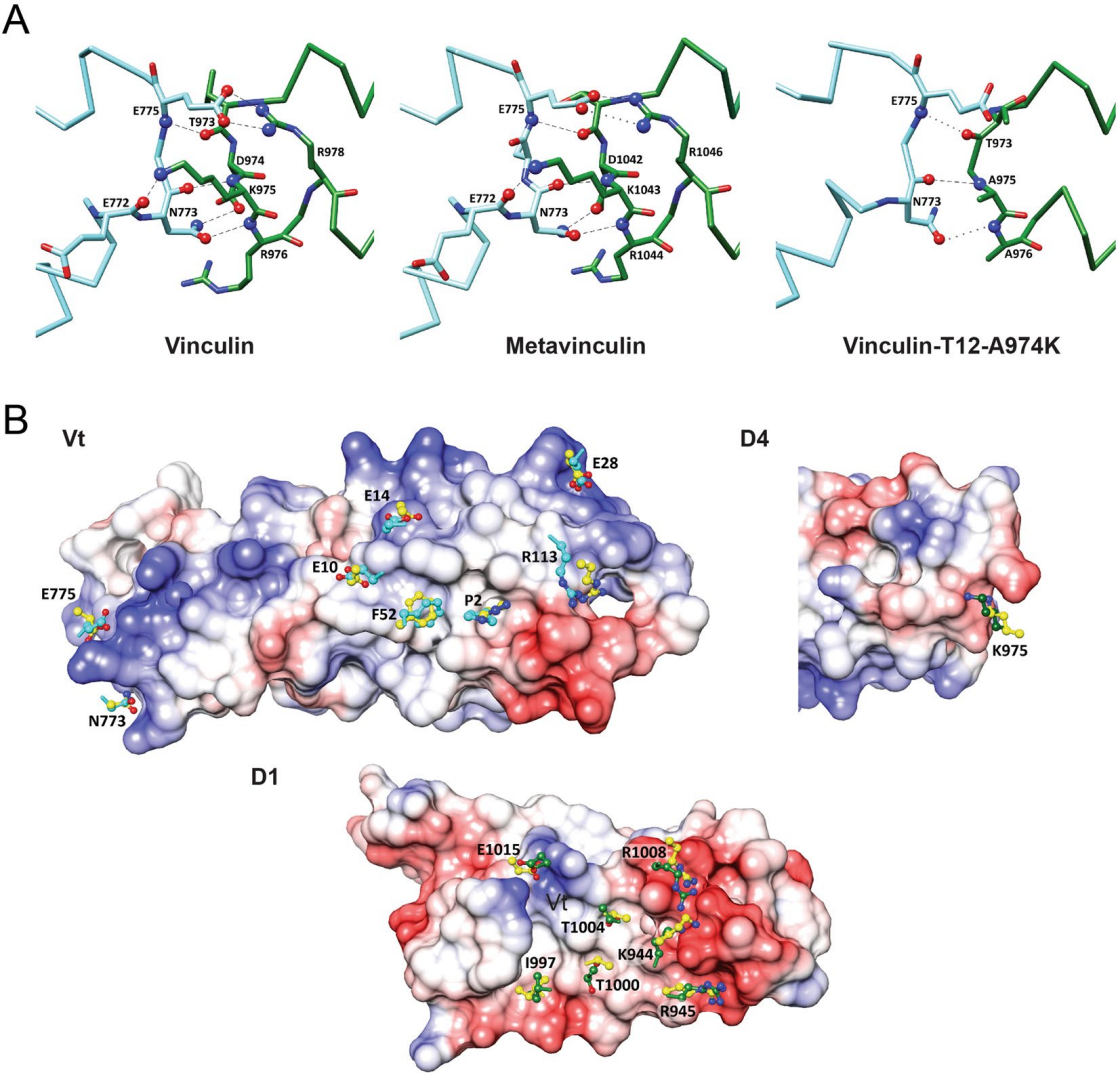
Internal interactions of the vinculin variants. (**A**) Hydrogen bonds at the D4-Vt interface. Vt is shown in dark green and D4 in light blue. Hydrogen bond contacts shorter than 3.5 Å are indicated by the dashed lines. Longer hydrogen bonds are indicated by the dotted lines. (**B**) Anchoring spots at the D1-Vt and D4-Vt interfaces of vinculin. The surfaces of the domains are colored by the electrostatic potential, red for negative, blue for positive and white for neutral. The computationally predicted anchoring spots are shown in yellow and the corresponding residues in the experimental structure are shown in dark green for Vt and light blue for D1 and D4. The predicted anchors include only side-chains, starting from atom Cβ, except for Thr and Ile. Cα atoms are shown as sticks.

In an attempt to study the structure of the active form of vinculin, we have subjected the vinculin-T12-A974K variant, which is considered to display the weakest head-tail interaction, to X-ray crystallography (Fig. 1D and Table S1 and PDB accession code: 6FMM). Surprisingly, the crystal structure we obtained for this variant was of a closed conformation, nearly identical to those of native vinculin^21^ and metavinculin^30^ (Fig. 1B–D). The structures show that all vinculin variants consist of five helix-bundle domains four of which (D1-D4) form the vinculin head (Vh) that is connected through a long, partially unresolved, linker (“hinge”) to the fifth helical bundle (tail domain; Vt). In the closed conformer, Vt appears as a rod whose two ends bind to head domains D1 and D4, via residues conserved in vinculin and metavinculin; a small additional contact is made with D3. The properties of the Vt-D1 and Vt-D4 interfaces differ considerably. The Vt-D1 interface is large (2085 Å^2^, averaged for the two independent polypeptide chains in structure 1TR2), and mostly hydrophobic; yet it is reinforced by hydrogen bonds between positive Vt residues and negative D1 residues. The Vt-D4 interface is considerably smaller, 696 Å^2^ (on the average) and largely electrostatic, positive on the Vt side and negative on the D4 side (Fig. 2).

The T12 and T12-A974K mutation sites (residues D974, K975, R976 and R978) map to the Vt-D4 interface and hit hydrogen bonds that stabilize this interface (T974-N773, K975-E772 and R978-E775). The loss of these hydrogen bonds also leads to lengthening of hydrogen bonds between back-bone atoms in T12-A974K (Table 1 and Fig. 2A). Hence, the Vt-D4 interaction in the vinculin mutants T12-A974K and T12 is likely to be considerably weaker than in wild type vinculin.

**Table 1:** Hydrogen bond interactions at the Vt-D4 interface as seen in the crystal structures of vinculin, metavinculin and vinculin-T12-A974K (Å).

vinculin^1^		metavinculin		vinculin-T12-A974K	
T973/O – E775/N	2.89 2.75	T1041/O – E775/N	3.26	T973/O – E775/N	3.65
D974/Oδ – N773/Nδ	3.04 3.04	D1042/Oδ – N773/Nδ	3.26		
K975/N – N773/O	2.84 3.01	K1043/N – N773/O	3.03	A975/N – N773/O	3.28
K975/Nζ – E772/O	2.35 2.29	K1043/Nζ – E772/O	3.02		
R976/N – N773/Oδ	2.94 3.03	R1044/N – N773/Oδ	3.01	A976/N – N773/Oδ	3.63
R978/Nh – E775/Oε	2.62/2.71^2^ 3.00/3.19^2^	R1046/Nh – E775/Oε	2.47		

^1^For vinculin two values are listed for the independent polypeptide chains in the asymmetric unit of structure 1TR2.

^2^bifurcated hydrogen bond.

We further analyzed the Vt-D1 and Vt-D4 interfaces of native vinculin by anchoring spot mapping^31^. An anchoring spot consists of a cavity on the surface of a protein (or protein domain) that binds a protruding amino acid side chain of another protein (or domain), and the mapping tool determines the location and binding energy of the protruding amino acid. Anchoring spots with ΔG ≤ 4 Kcal/mol were shown to correspond to very strong experimental hot spots^31^. Table 2 lists thirteen strong anchoring spots in the Vt-D1 interface, these are presented in Fig. 2B. Seven of them (K944, R945, I997, T1000, T1004, R1008 and E1015) are Vt residues that bind in cavities on the surface of D1, and the other six (P2, F4, E10, E14, E28 and R113) are D1 residues that bind in cavities on the surface of Vt. The Vt-D4 interface includes only three strong anchoring spots, which consist of D4 residues N773 and E775 and Vt residue K975. Anchor residues N773 and E775 form hydrogen bonds to the side chains of D974 and R978, respectively; mutation of these residues to alanine would significantly weaken the Vt-D4 interactions in vinculin-T12 and vinculin-T12-A974K compared to native vinculin. In T12-A974K the additional positive charge augments the positive potential on the Vt side of the Vt-D4 interface. The structure, however, shows that K974 does not form a hydrogen bond with D4; rather, hydrophobic contacts between the CH_2_ groups of K974 and the side chain of N773 are observed. The number of contacts involving K974 exceeds the number of contacts that A974 can make and together with the augmented positive potential it is possible that the Vt-D4 contact in vinculin-T12-A974K is stronger than in vinculin-T12.

**Table 2:** Anchor residues that correspond to calculated low ΔG (in Kcal/mol) anchoring spots.

The Vt-D1 interface		The Vt-D4 interface	
Residue	ΔG^1^	Residue	ΔG
K944	−4.0*	N773	−3.5
R945	−5.8*	E775	−3.2*
I997	−3.6	K975	−4.1*
T1000	−3.1		
T1004	−3.3		
R1008	−4.9*		
E1015	−3.2		
P2	−3.9		
F4	−3.8		
E10	−3.3		
E14	−3.9		
E28	−8.8		
R113	−3.5*		

^1^The ΔG values are averages of anchoring spots calculated for the two independent polypeptide chains in the asymmetric unit of structure 1TR2.

^*^Cases of which anchoring spot was detected for only one of the chains.

### Metavinculin and vinculin-T12-A974K occupy a larger conformational range compared to vinculin and vinculin-T12

Considering that crystal structures, cryogenically frozen before analysis, often reflect only a subset of possible protein conformers^32^, we were wondering whether the closed conformation displayed by all vinculin variants, is indeed the only occurring stable state of these proteins in solution. To address this issue we applied the ion mobility-mass spectrometry (IM-MS) approach, which enables the characterization of the ensemble of conformational states of a system in aqueous conditions^33,34^. In this method, the time it takes a protein to traverse a weak electrical gradient in a gas-filled chamber is measured. The drift time is proportional to the number of gas molecules the protein collides with along its path, so that proteins of similar mass, but with less compact conformation will traverse the IM chamber more slowly. As the number of collisions is proportional to the surface area of the protein, a rotationally averaged collision cross section (CCS) value can be calibrated by measuring the drift time of proteins and protein complexes of known shape and structure.

**Figure 3:**
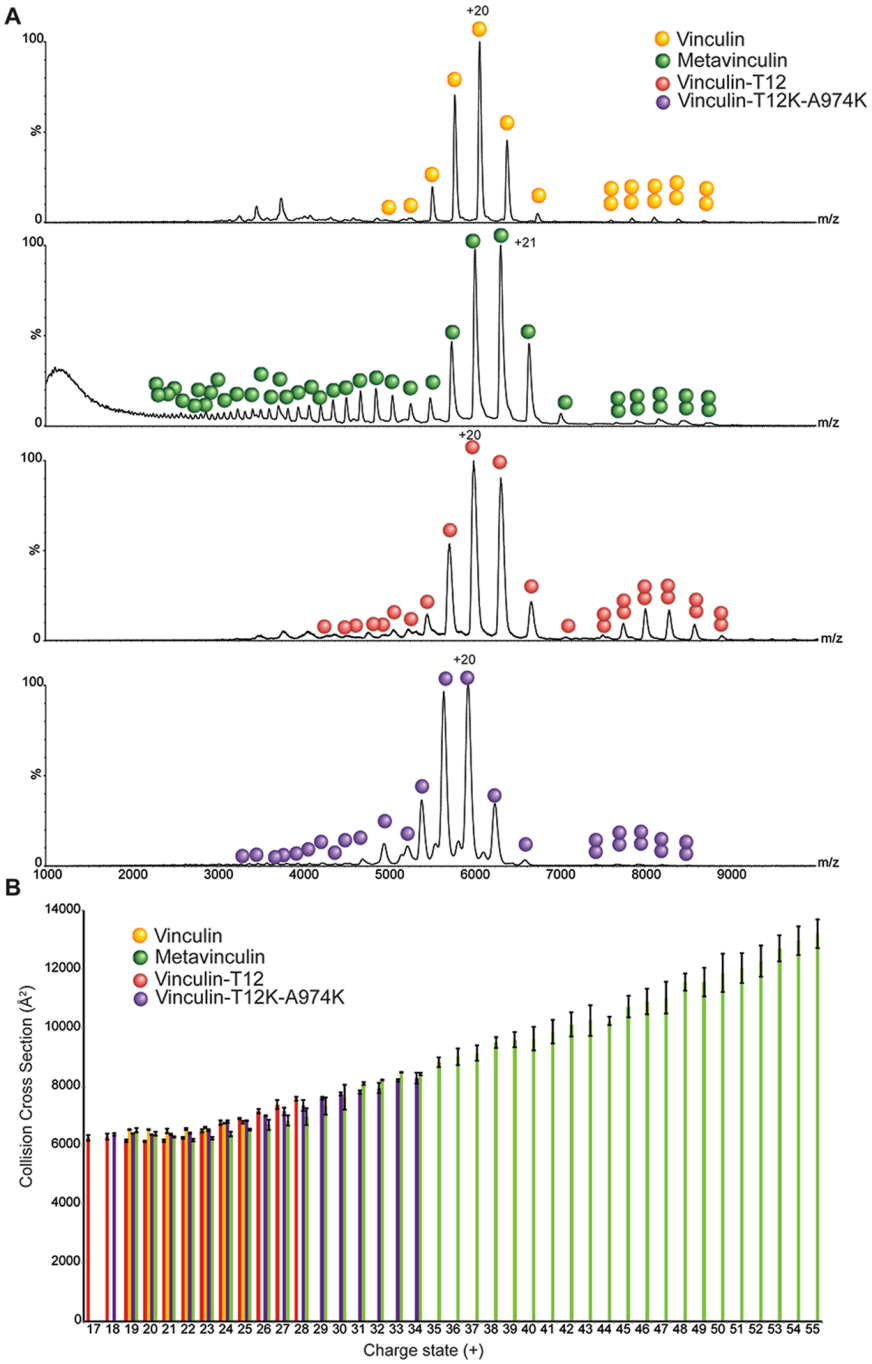
Vinculin variants exhibit altered conformational plasticity. Vinculin, metavinculin, vinculin-T12 and vinculin-T12-A974K were subjected to IM-MS analysis and their collision cross section was calculated. (**A**) Representative MS dimension projections of the four vinculin variants emphasizing the variability in charge state distribution. Both vinculin-T12 and to a larger extent metaviculin, display a wide distribution of charge states (29^+^–55^+^) compared to vinculin and T12. This heterogeneous, highly charged population indicates the existence of extended conformers. (**B**) Experimental CCS values. The high charge states of metavinculin display a significantly larger CCS value compared to the lower charge states (17^+^–28^+^) or to the CCS values obtained for vinculin and vinculin-T12. Error bars represent the standard deviation of 3 different wave heights.

Using this approach, we first examined vinculin. An IM–MS spectrum of vinculin shows a series of charge states separated in both m/z and drift time dimensions. The m/z projection of the IM-MS spectrum indicated 7 major charge states centered at 5,700 m/z (Fig. 3A) corresponding in mass to monomeric vinculin. A calculated CCS value of 6,531 ± 7 Å^2^ was obtained from the IM-MS measurements for the most intense charge state (20+) (Fig. 3B). Theoretical CCS for human vinculin gave a value of 6,460 Å^2^, which fits well with the measured CCS value. Overall, these results suggest that in the absence of external perturbation, vinculin, in solution, exists entirely in the closed, head-to-tail packed, conformation.

Examination of metavinculin recordings indicated that it includes a considerably higher number of charge states, with a much broader distribution than that of vinculin (Fig. 3). It was previously shown that the number of charges a protein can accommodate during the electrospray ionization process is correlated with its surface area^35,36^. Therefore, extended protein conformations, as well as partially or fully unfolded states, will give rise to higher charge states compared to those of the protein’s compact folded structure. Consequently, the larger number of charges observed for metavinculin suggests that in addition to the folded state, additional populations, with larger surface area, exist. This assumption was then validated by the CCS measurement. Metavinculin yielded a wide range of CCS values, which could be roughly divided into two groups. One population (charge states 19^+^–24^+^) displayed CCS values similar to vinculin, while the second population (charge states 25^+^–56^+^) exhibited much larger CCS values, up to 13,200 Å^2^, indicating the existence of a large population of molecules (in the order of ~40%) with extended or unfolded states (Fig. 3B). The conformational flexibility of metavinculin is likely to originate from its 68 amino acid insert residing between the tail and the hinge domains. However we cannot rule out the possibility that the unfolded states of metavinculin were generated during the isolation and purification of the protein. Nevertheless, this region exhibits no electron density in the crystal structure, suggesting that it is indeed disordered. Taken together, these results suggest that the inserted segment of metavinculin modifies the dynamics of the head-to-tail lock, enabling metavinculin to form a more heterogeneous and extended set of conformations than vinculin.

We then set out to examine whether vinculin-T12 and vinculin-T12-A974K, in solution, are constitutively open, as predicted, or closed, as suggested by X-ray crystallography. The m/z projection of vinculin-T12 and vinculin-T12-A974K showed that they exhibit 11 and 16 charge states, respectively (Fig. 3A). This charge state distribution is considerably narrower than that of metavinculin, however it is wider than that of vinculin, suggesting that vinculin-T12 and to a larger extent vinculin-T12-A974K, possess a somewhat greater structural heterogeneity than vinculin. The experimental CCS value for the highest charge state peak corresponded to 6,137 ± 29 Å^2^ for vinculin-T12 and 6,359 ± 9 Å^2^ for vinculin-T12-A974K. Thus, despite the elimination of three hydrogen bonds that anchor the head to the tail, these domains remain in contact in vinculin-T12 and vinculin-T12-A974K, in consistence with the crystal structure.

### Disruption of the head-to-tail interaction of vinculin is a two-step process

To explore the externally-induced conformational transition, associated with vinculin activation, we exposed the four vinculin variants to the Collision-Induced Unfolding (CIU) approach, which couples collisional molecular perturbation with ion mobility measurements^37,38^. In this this type of experiment, the energy in the collision cell is elevated in a stepwise manner, causing protein activation that may consequently induce conformational change. The collision voltage at which the transitions between conformations occur, the mode of the transition and the size of intermediates, generate a characteristic unfolding trajectory of the protein. Specifically, upon collision activation we expected that the head-to-tail constrains of the four vinculin forms will be resolved, yielding distinct CIU characteristics.

Considering that ions with low charge states usually preserve the native protein structure^39,40^, we have reduced the number of charges on the proteins by utilizing triethylammonium acetate. The 17^+^ charge state, which for all vinculin forms corresponds to the closed compact head-to-tail conformation, has been selected for CIU analysis. We then took advantage of the fact that charge reduction also induces separation between protein peaks of similar mass, as their total mass is now divided by a smaller number of charges, to simultaneously measure the CCS plots of a mixture of vinculin forms, while avoiding overlapping peaks and experimental variability. This approach enabled direct comparison between the different vinculin variants, allowing identification of even subtle structural changes, as effects of needle positioning and needle–needle variations that influence the measurements were eliminated^41^. Towards this, experiments were performed in two series, one containing vinculin, metavinculin and vinculin-T12, and the other containing vinculin, metavinculin and vinculin-T12-A974K.

**Figure 4:**
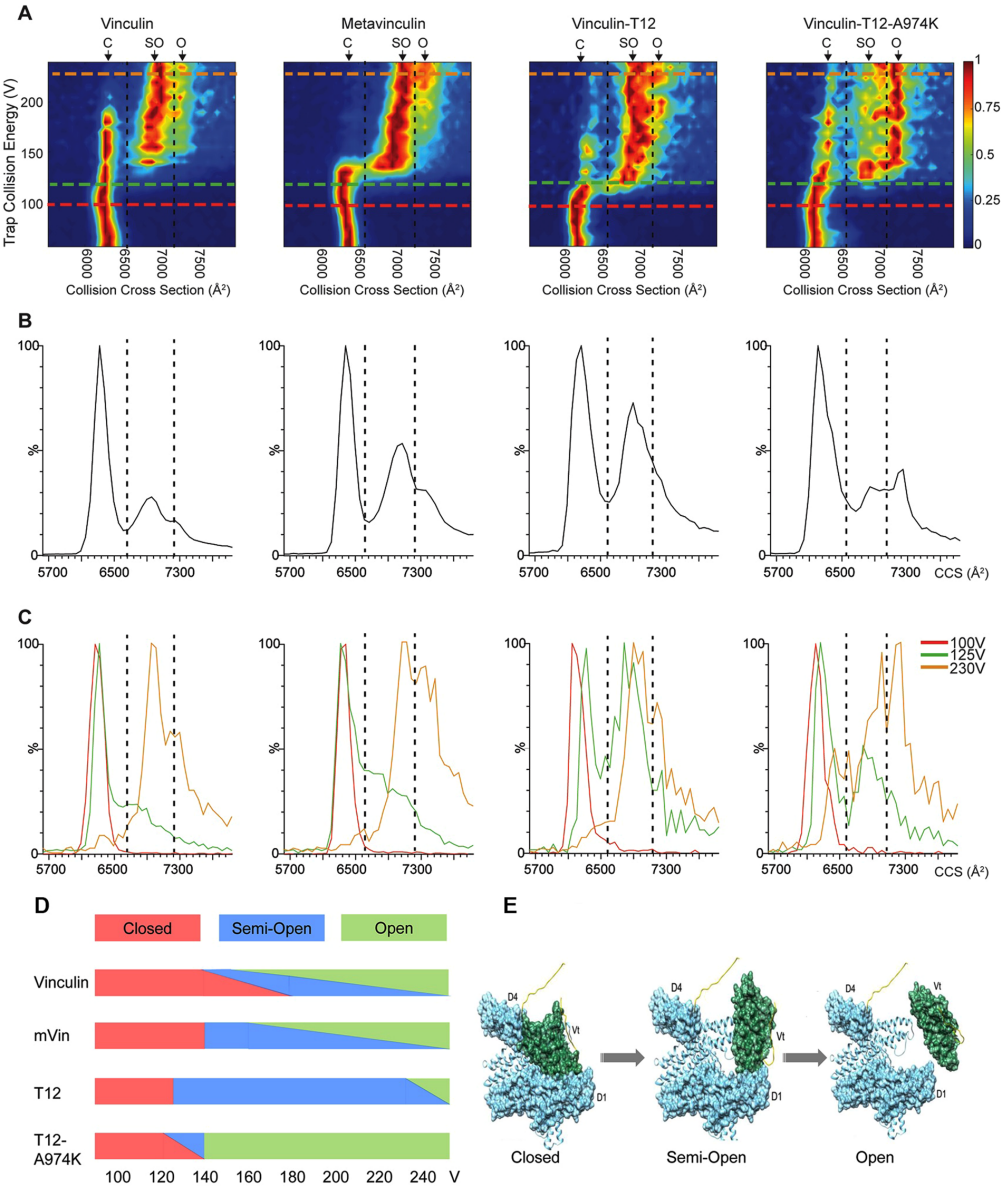
Collision induced conformational transitions in the four vinculin variants indicate the existence of a two-step opening mechanism. (**A**) CIU contour plots of the 17^+^ charge state of vinculin, metavinculin, vinculin-T12 and vinculin-T12-A974K, where CCS is plotted against collision voltage. Colors denote signal intensity as indicated. A similar unfolding pattern involving the two transition states from closed (C) to semi-open (SO) and consequently to open (O) conformations is observed for all proteins. Nevertheless, they differ in the energy threshold that is required for the transitions as well as in the range of voltages they dwell in each state. For clarity dashed lines were added. (**B**) Average CCS plots obtained for all measured collision voltages, reflecting the relative abundance of the three discrete states. (**C**) Superposition of CCS profiles obtained at collision energies of 100, 125 and 230 V emphasize the differences in the transitions state threshold of the various vinculin forms. Images were generated from a representative experiment out of 4 repetitions. (**D**) A graphic representation of the data shown in panels (A) and (B). This illustration emphasizes the gradual transition of the C form of vinculin to SO. The transition of vinculin between the C to SO states is gradual, in contrast to the more abrupt shift of the other vinculin forms. Moreover, the transition of the two T12 forms occurs at a lower acceleration voltage, compared to vinculin and mVin. In addition, the apparent instability of the SO conformer of T12-A974K, compared to all other forms, and, in particular, to T12 is reflected. (**E**) Structural models of the proposed transitions between closed, semi-open and open conformations of the different vinculin variants.

We first examined the unfolding properties of native vinculin. The CIU contour plot shown in Fig. 4A, reflects the changes in the CCS values as a function of collision voltages. At low collision voltages, vinculin has an average CCS value of 6,266 ± 35 Å^2^, which is in agreement with the calculated CCS for the closed compact conformation of vinculin (6490 Å^2^) we therefore termed it the C (closed) state (Table 3). This configuration persists until reaching a collision voltage of 180 V, however, already at 140 V a transition occurs to a more extended, most likely “semi-open” conformation (state SO), giving rise to a CCS value of 6,970 ± 31 Å^2^. The transition of vinculin between states C and SO is a gradual process, whereby the increase in acceleration voltage leads to decreased levels of the compact form and a reciprocal increase in the SO state. An additional structural transition is observed at collision energies larger than 180 V, exhibiting a further increase in the CCS value, reaching 7,268 ± 15 Å^2^. This conformation persists when the maximal voltage limit is reached; we therefore termed it the “open structure” (state O). Overall two distinct and well defined ensembles of conformations are detected in addition to the most compact protein configuration. For comparison with the other vinculin forms, we use a simple (C, SO and O) nomenclature for these conformational transition families.

**Table 3:** Experimental collision cross measured from collision induced unfolding experiments. Values represent averages and standard deviations of 3 independent experiments in Å^2^.

Protein\Conformation	C	SO	O
Vinculin	6266 ± 35	6970 ± 31	7268 ± 15
Metavinculin	6281 ± 32	7056 ± 30	7363 ± 56
Vinculin-T12	6108 ± 38	6847 ± 28	7173 ± 125
Vinculin-T12-A974K	6164 ± 28	6850 ± 18	7279 ± 5

The SO peak is clearly separate from the C state peak, suggesting that it represents a discrete state, distinct from the closed conformer (Fig. 4A–C). Therefore, in order to relate the measured collision cross sections to the structural changes that vinculin undergoes, we calculated the CCS for several in-silico models in which Vt of native vinculin was shifted away from Vh to a variety of locations. There was no one-to-one correspondence between the models and the calculated CCS values, namely, different models had similar CCS values. Yet, the calculated CCS increased as the distance between Vt and Vh increased and it reached 8,325 Å^2^ when Vt residue 875 was placed 125 Å away from D4 residue 836, a distance that approximately spans the length of an extended hinge. Figure S1 presents selected models with calculated CCS that match the experimental CCS values (Table 3). In the C state, the head (Vh) domains D4 and D1 bind to the tail (Vt) domain. The SO state of vinculin, in which the interaction of Vt with either D4 or D1 is disrupted, is represented by two possible models, SO1 and SO2, respectively (Supplementary Figure 1). The CCS calculations do not distinguish between the two options but the fewer and less energetic anchoring spots (see Table 2) at the significantly smaller interface of Vt-D4 compared to Vt-D1 strongly suggest that the Vt-D4 interface disrupts more readily than the Vt-D1 interface. Hence, the SO state likely corresponds to a conformation in which Vt is still in contact with D1 but not with D4. The CIU results indicate that C and SO conformers co-exist for a long range of collision voltages suggesting structural reversibility between the closed and semi-open conformations. The calculated CCS for an open vinculin, in which Vt is detached from Vh but does not move far away from it (Supplementary Figure 1), is in correspondence with the O state. The CIU peak for the open state appears only after the closed conformer disappears and it merges with the SO state, suggesting continuous and reversible transition from semi-open to open conformers but not from closed to open conformers.

Inspection of the CIU plot of mVin, shows a similar pattern of two transition steps. Detailed analysis, however, reveals that unlike vinculin, the conformational transition from C to SO in mVin is abrupt; occurring at ~140 V, whereby the C conformer of mVin disappears and the SO state simultaneously appears. This observation reflects different transition dynamics in mVin that can be attributed to the longer Vh-Vt linker. Thus, once the Vt-D4 interface is disrupted the longer linker hampers reversibility and affects the equilibrium between the SO and O conformers, explaining the more pronounced presence of the O state in mVin. The ease of the C-to-SO transition in mVin is also in line with the accumulation of high levels of unfolded molecules in solution (Fig. 3).

Interestingly, as seen Fig. 4A–C, vinculin-T12 shifts between the compact C state and SO much earlier than vinculin, at the relatively mild conditions of 120 V. Since the T12 mutations at the Vt-D4 interface weaken this interaction, the observed early transition from the C to the SO state further supports our conclusion that SO represents a semi-open conformation in which Vt remains bound to D1 but detaches from D4. Hence, because of the weaker Vt-D4 binding, less energy is needed in order to induce the transition from closed to semi-open T12. This also explains why the C of T12 state does not persist at higher collision voltages in comparison to vinculin. Interestingly, the C state of vinculin-T12-A974K persists longer than that of the T12 variant, but the SO state, detected at low voltage just like in T12, is considerably less stable, and occurs for only a narrow range of collision voltages, before the next transition to the O state takes place. This higher stability of the C state in vinculin-T12-A974K (compared to vinculin-T12), is attributable to the hydrophobic contacts between the CH_2_ groups of K974 and D4, as seen in the crystallographic structure. Stabilizing electrostatic interactions between K974 and D4 are not observed in the crystal structure of vinculin-T12-A974K, however, they can occur in solution or under the MS experimental conditions.

Taken together, these results show that all forms of vinculin exist in a closed conformation in the absence or at low level (<120 V) of collision voltage (Fig. 4D). Mutations that disrupt the head-to-tail binding (vinculin-T12 and vinculin-T12-A974K) can affect both the C-to-SO and SO-to-O transitions. The unexpected effect of the mVin insert indicates that the extended unstructured hinge region in mVin affects the C-to-SO transition. These results are in line with the crystal structures that show two-end binding of Vt to the D1 and D4 domains of Vh, forming two separate and apparently partially independent head-to-tail links. In accordance with this assumption, the CIU profiles indicate that the opening of the different vinculin variants is a two-step process, switching from closed to semi- and fully-open states (Fig. 4E).

### The differential effects of vinculin and its variants on focal adhesion morphology and dynamics

**Figure 5:**
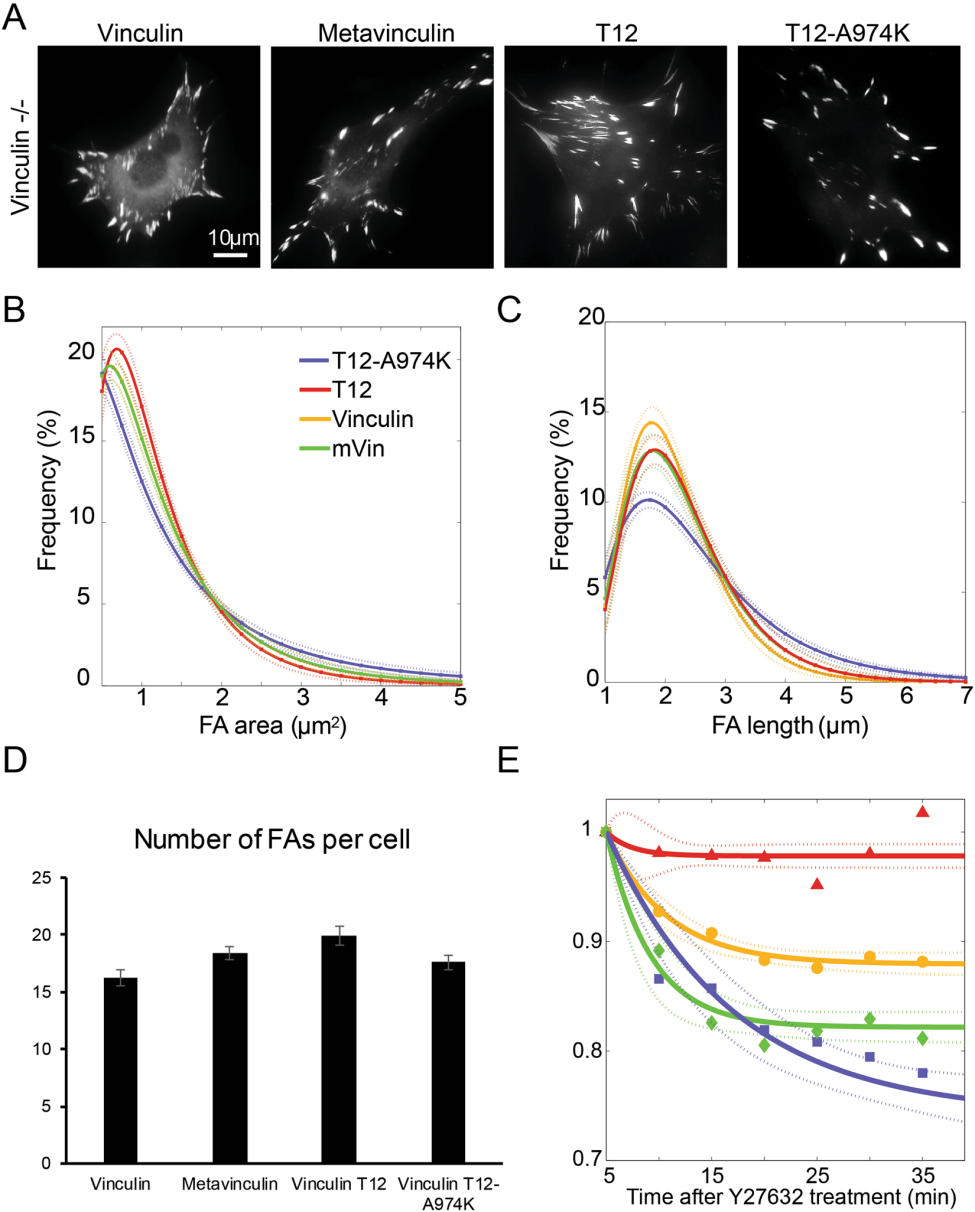
Vinculin variants have distinct effects on FA morphology in vinculin −/− MEF cells. (**A**) The fluorescently-tagged vinculin variants localize to FAs. Scale-bar is 10 µm. (**B**–**D**) Quantification of focal adhesion properties in vinculin null MEFs, expressing the four vinculin variants. Solid curves represent best fits to log-normal distribution profiles with 95% confidence bounds (dashed lines). Error bars are standard deviation. (**E**) Time response of the different vinculin variants to Y27632 treatment in vinculin null MEFs. Whereas T12 completely (vinculin null MEFs) stabilized FAs, mVin and T12-A974K had the opposite effect of increasing FA instability. Solid curves are best fits to an exponential decay profile with 95% confidence bounds (dashed lines). Mean focal adhesion intensity was normalized with respect to its value just upon addition of 10μm Y27632 at time = 0, and corrected for bleaching.

The fact that the four vinculin variants studied here contain the same functional domains yet they greatly differ in their head-to-tail lock strength, enabled us to explore the role of head-tail interaction in FA formation, maturation and dynamics. To this end, we expressed each of the variants in two cellular systems, namely vinculin-null mouse embryo fibroblasts (MEF), where the transfected proteins are the only vinculin proteoforms present (Fig. 5) and HeLa cells (Figure S2), which co-expresses endogenous wild-type vinculin. Representative images and the corresponding quantification of FA features, based on quantification of approximately 1,000 FAs in each experimental group, revealed highly significant differences in FA area (Fig. 5B and Supplementary Figure 2B), length (Fig. 5C and Supplementary Figure 2C), dynamics (Figure S3) and number (Fig. 5D and Supplementary Figure 2D) which were consistent in both the HeLa cells (Supplementary Figure 2) and the vinculin null MEFs (Fig. 5). In both cell types, WT vinculin expression correlated with enrichment of small (<1µm^2^) and short (<2 µm) FAs, while T12 and, especially T12-A974K, being the largest and longest. The translocation of FAs in the different cells was variable, but consistently, cells containing vinculin were more dynamic than those expressing T12, T12-A974K and mVin (Supplementary Figure 3). Interestingly the number of FAs was essentially the same in cells expressing each of the vinculin variant, suggesting that the different variants affect differentially FA growth and stability and not FA initiation.

To explore the relevance of the CIU data for the force dependence of FA formation we exposed the vinculin null MEFs, expressing each of the fluorescently-tagged vinculin variants to moderate levels of the Rho-kinase inhibitor Y27632, and monitored, by live cell microscopy, the FA-associated fluorescence, enabling us to determine the differential effects of each of the variants on the force-dependence of FAs. As shown in Fig. 5E and Supplementary Figure 2E, vinculin expressing cells displayed a marked decline in FA fluorescence, with an initial slope of 1.2%/min while the expression of T12 completely stabilized FAs, supporting the notion that the T12 variant, in cells, is in the semi-open state, irrespective of actomyosin contractility. Interestingly, both mVin and T12-A974K, induced an increased sensitivity of FAs to Y27632, which is correlated to their tendency to readily proceed to the open state. Taken together, the transition profiles of the four vinculin forms, from closed conformation to semi-open to open bear a compelling relevance to FA formation, overall structure, dynamics and mechanosensitivity. The “tunable”, gradual transition of WT vinculin from closed to semi-open conformation, may account for the formation of small and dynamic adhesions, compared with the large and more sessile adhesions formed in the presence of the two T12 mutants, which, apparently require milder mechanical perturbation for full activation. Finally, the tendency of mVin and T12-A974K to readily proceed from the semi-open to the open state correlates with FA instability, suggesting a possible mechanism whereby excessive force downregulates FA durability.

## Discussion

Our objective, in this work, was to monitor the conformational states of vinculin following the exposure to external perturbation. To determine the contribution of the key sites, which are expected to regulate the head-tail interactions, we compared vinculin with three variant molecules. Surprisingly, comparison of the new T12-A974K structure with the published structure of WT vinculin and of mVin indicated that despite the loss of 4 hydrogen bonds and the weakening of the remaining ones, the crystal structure clearly displayed a closed conformation. This can possibly be attributed to the strong binding of the head domain D1, which directs the other end of the rod-like Vt to bind to D4.

Being aware of the possibility that the crystal structure does not always represent the proteins’ dynamics in solution, we have subjected vinculin and the three variants to IM-MS analysis, which enables the derivation of experimental CCS values that are related to the surface area of a protein in solution^33,34^. The IM-MS derived CCS values of vinculin, T12, T12-A974K and mVin indicated that these proteins exist in a closed conformation, supporting the X-ray crystallography results. T12-A974K and to a larger extent mVin, however, also possessed a population of conformers exhibiting larger CCS values, suggesting that the proteins exist, to some degree, in a partially unfolded conformation. In the case of mVin this is probably caused by its unstructured long insert positioned between the tail and the flexible hinge region. As suggested below, although the dominant form of the vinculin variants is the closed conformation, the extended conformations of T12-A974K and mVin harbor biological significance.

We next used the CIU approach to assess the differential structural properties of the vinculin forms, in response to acceleration energies^37^. These experiments revealed several unexpected features of the collision-induced conformational transitions of the four studied molecules. An important observation is that for vinculin and each of the variants, there were three discrete conformational states. The first corresponds to the closed (C) conformation, the second, to a semi-open (SO) state, and the third – to an open (O) state. Calculated CCS values, based on model structures, suggest that an O state can be reached, in which the head and tail domains are detached, while maintaining the folded tertiary structure of each domain intact (Fig. 4E). Moreover, the distinct states appear to be stable species that exist for wide ranges of acceleration voltages, with only one exception, namely the semi-open state of T12-A974K, which readily proceeded to the open state (Fig. 4D). Thus, a modular path of structural transitions exists between the C and O forms of the vinculin variants.

Comparing the four tested molecules, some interesting insights should be emphasized (see Fig. 4D): (i) the C to SO transition appears to be tightly regulated by the tail-D4 interaction, hence, the two T12 mutants require lower acceleration voltage (120 volts) to undergo this transition in comparison to WT vinculin and metavinculin. (ii) The mode of transition of vinculin from the C state to the SO (between 140–180 V), is gradual and possibly reversible. Unlike vinculin, the three vinculin variants undergo more abrupt transitions between the C and SO states. (iii) An intriguing observation made here is that the SO state in vinculin, mVin and T12 is relatively stable, while in T12-A974K the SO state is highly unstable. Thus, although the C to SO transition of T12 and T12-A974K is initiated at the same acceleration voltage, T12-A974K rapidly transforms to a stable O state. Interestingly, these findings are supported by recent a molecular simulation study that indicated that vinculin activation coincide by two steps^42^.

Considering this information concerning the conformational transitions of the different molecules, we have tested the differential effects of vinculin, mVin and the two T12 variants on FA size, dynamics and force dependence. The live cell examination provided compelling functional information. It indicated that the size of FAs is clearly higher in cells expressing either T12 or T12-A974K, which both, undergo transition to the SO state under the mildest conditions, in the CIU experiment (Fig. 4). These results also suggest that the SO state is the active conformation of vinculin that drives FA growth. Another interesting observation is that T12 expression in cells converts FAs to force independent, while T12-A974K exerts a nearly opposite effect - namely its destabilization of FA in Y27632 cells (Fig. 5). This striking difference between the two molecules, which differ in only one amino acid, can be attributed to the apparent instability of the SO state and “premature” formation of a fully open vinculin in T12-A974K (Fig. 4A). The possibility that open vinculin might destabilize FAs, is in line with the observation that mVin, which is partially open in solution, also destabilizes FAs.

Taken together, the results presented here indicate that vinculin appears to have a very stable closed conformation, which can gradually undergo a transition to the active, semi-open state, driven by actomyosin contractility. This gradual “unlocking” of the closed conformation appears to depend on the force applied to the Vt-Vh(D4) interface, which can break the four hydrogen bonds present at this site (T974-N773, K975-E772 and bifurcated R978-E775). The elimination of these four bonds in the two T12 mutant molecules leads, not only to unlocking at lower activation level, but also to loss of the force-tunable activation process, and acquisition of a sharp transition from close to semi-open state. Interestingly, metavinculin started to undergo C-to-SO unlocking under the same level of mechanical perturbation as vinculin, but the process was abrupt, suggesting that the extra stretch of 68 amino acids at the hinge-tail interface affects the dynamics of the Vt-D4 interface. It is noteworthy that in solution, a significant proportion of metavinculin was already in the open state, which raises an intriguing question, whether the fully open vinculin or metavinculin can trigger or stabilize focal adhesions. We do not have direct information on the activity of the fully open vinculin and, in fact the results presented here support just the opposite, namely that the open forms (metavinculin and T12-A974K, with its highly unstable semi-open state) render them more sensitive to actomyosin inhibition.

## Methods

### Cell culture and reagents

HeLa JW and vinculin-null MEFs were grown in DMEM (Gibco, Grand Island, New York) containing 10% FCS and 100 U/mL PenStrep (Biological Industries, Beit Haemek, Israel) at 37 °C in a 5% CO2 humidified atmosphere. ROCK inhibitor Y27632 (Sigma-Aldrich, St. Louis, MO) was used at a concentration of 10 µM.

### Overexpression of vinculin, metavinculin, vinculin-T12-A974K and vinculin-T12

HeLa and vinculin-null MEF cells were seeded onto optical glass bottom 24 well plates (MatTek, Ashland, MA) and then transfected with the different vinculin variants. HeLa cells were transfected using jetPEI (Polyplus-transfection, France), while vinculin-null MEF cells were transfected using either Lipofectamine 2000 or TurboFect (both Thermo Fisher Scientific, Waltham, MA). All transfections were done according to the manufacturers’ protocols. The vinculin variants were conjugated with GFP (except for vinculin-T12-A974K which was conjugated with mCherry) as described in Zamir *et al*.^43^.

### Immunofluorescence microscopy and image analysis

Images and time lapse movies were acquired using a Deltavision Elite (GE Healthcare/ Applied Precision, USA) system mounted on an inverted IX71 microscope (Olympus, Japan) connected to a Photometrics CoolSNAP HQ2 camera (Photometrics, Tucson, AZ). The system was running SoftWorX 6.1.3. Pictures were taken with an Olympus UIS2 BFP1 60× oil PlanApoN objective with a numerical aperture of 1.42 (Olympus, Japan). Quantification of FA area, length and fluorescence intensity was performed using a custom code written in Matlab (MathWorks, Natick, MA) after correcting for fluorescence bleaching.

### Protein purification

The genes encoding WT Vinculin (2-1066), metavinculin (68 AA insertion following residue 915 of the wt sequence) and Vinculin-T12 (with the following mutations: D974A, K975A, R976A and R978A) were each cloned into pET28_TEVH. All three proteins were produced an purified in the same manner as follows: the plasmids were transformed into *E. coli* BL21(DE3) and a 5 L culture was grown in LB medium at 37 °C until mid-log phase. Protein expression was induced by the addition of 0.2 mM IPTG and allowed to continue growing at 15 °C ON. After harvesting the cells by centrifugation, the cell pellet was lysed using a cell disrupter (Constant Systems) in 100 ml buffer containing: 50 mM Tris pH = 8, 0.5 M NaCl, 20 mM Imidazole, protease inhibitor cocktail set III (Calbiochem), Dnase, 1 mM PMSF and Lysozyme. The insoluble material was removed by centrifugation at 26,000 g for 30 min. The supernatant was filtered and loaded onto a Ni Column (HiTrap_chelating_HP, GE) equilibrated with lysis buffer. The protein was eluted in one step with the same buffer containing 0.5 M Imidazole. Fractions containing Vinculin were loaded onto a size exclusion column (Hiload_26/60_Superdex200, GE), equilibrated with 50 mM Tris pH 8 and 50 mM NaCl. The pooled fractions containing Vinculin were injected into an anion exchange column (Tricorn Q 10/100 GL, GE) equilibrated with 50 mM Tris pH = 8. Pure Vinculin was eluted with a linear gradient to 1 M NaCl. The pure protein was flash frozen in aliquots using liquid nitrogen and kept at −80^o^ C. For purification of vinculin-T12-A974K, Cultured Sf9 insect cells at a density of 1.5 to 2.5 mio/ml were infected with P1 virus and incubated for ~27 hours at 27 °C. Expressing cells (green color) were harvested and lysed in 20 mM Tris-HCl (pH = 7.5), 0.4 M NaCl, 1 mM DTT, 0.01% Triton, PMSF, and PI cocktail. The protein was purified over a nickel affinity column and eluted in presence of 3 C protease. Vinculin was further purified in 20 mM Tris-HCl (pH = 7.5), 0.1 M NaCl and 1 mM DTT using a size exclusion column (Superdex 200 Increase 10/300 GL). Protein concentration was determined, aliquoted and stored at −80 °C.

### Crystallization

Purified vinculin T12-A974K (~6 mg/ml) was crystallized using the vapour diffusion, sitting drop method at 4 °C. First crystals emerged from initial screens and were used for microseeding, yielding new crystals gained against a reservoir solution consisting of 15% (w/v) polyethylene glycol (PEG) 4 K, 0.2 M calcium acetate, 0.1 M Tris-HOAc (pH 8.5), and 15% ethylene glycol (EG) for cryoprotection. A total of 70 crystals were fished and flash frozen in liquid propane.

### X-ray diffraction data collection

Data sets were collected on frozen crystals on the Xo6DA (PXIII) beamline at the Swiss Light Source of the Paul Scherrer Institute on a PILATUS 2 M detector (Dectris). Crystals diffracted to a resolution of ~2.9 and belonged to the trigonal space group P3121 (a = b = 97.81, c = 233.77, α = β = 90, γ = 120).

### Structure determination and refinement

The data were indexed, integrated and scaled with XDS. Molecular replacement was done with Phaser using a vinculin WT (PDB code 1TR2) search model. Model building was performed with the program Coot. Initial refinement was done with Refmac and finalized with Phenix. Rigid body refinement cycles with data from 20.0 to 3.0 Angstrom were alternated with manual model building and the R/R_free_ values were monitored throughout this process. The final model shows R/R_free_ values of 25% and 30.6%, good geometry and almost no residues in disallowed regions of the Ramachandran plot.

### Computational anchoring spots mapping and molecular modeling

We used a modified version of ANCHORSmap^31^, which includes threonine and proline anchoring spots mapping, to detect anchoring spots on the surface of native vinculin. Anchoring spots are energetically favorable binding locations of single amino acid side chains. Each anchoring spot consists of a binding cavity on the surface of a protein, bound to an amino acid side chain. The procedure detects small cavities on the surface of a protein, scatters thousands of amino acid probes near the cavities and determines optimal probe positions. The binding ΔG of the optimally posed probes are calculated employing a semi-empirical scoring function that includes van der Waals and solvation energy terms and an electrostatic energy term corrected for the dielectric shielding exerted by an approaching protein. In this study the positions of low ΔG anchors (ΔG ≤ −3 Kcal/mol) were compared to the X-ray structure of human vinculin (PDB code 1TR2), searching for anchors that closely corresponded to observed binding sites. The UCSF-Chimera software^44^ was used for structure analyses, anchoring spots visualization, preparation of the semi-open and open models of vinculin and preparation of Figs 1, 2, 4 and S1.

### Ion-mobility mass spectrometry

All ion mobility measurements were recorded on a Synapt G1 HDMS system (Waters Corp., UK) with a traveling-wave ion mobility device as described in detail elsewhere^45^. Proteins were first buffer exchanged into 200 mM ammonium acetate, pH = 7.6 (Sigma) using a Biospin 6 column (Bio-Rad) and their concentration was adjusted to a final concentration of 10 µM. Typically, protein aliquots of 2–3 µl were injected via a gold coated borosilicate capillary via a nano-ESI ion source. Acquisition parameters were as following- capillary voltage−1.25 kV, source temperature−25 °C, sampling cone−17 V, extraction cone−1.1 V, trap and transfer collision energy−5 V. For IMS wave velocity of 250 m/s and wave heights of 8–10 V were used. IMS calibration for conversion of drift times to collision cross section values was done as previously described^46^. Experiment was repeated three times. Data presented is from a representative experiment and is an average of three wave heights−8, 9, 10 V.

### Collision induced unfolding (CIU) assays

Collision induced unfolding experiments were performed on a Synapt G1 HDMS system (Waters Corp., UK). Initially, proteins were buffer exchanged into 200 mM ammonium acetate pH = 7.6 supplemented with 40 mM trimethylamine (TEA). The latter was added in order to induce charge reduction and avoid a large number of overlapping peaks. Experiments were done in 2 series combining three proteins at once to reduce experimental variability. The first series included vinculin, metavinculin and vinculin-T12, while the other included vinculin, metavinculin and vinculin-T12A974K. Collision induced unfolding was performed as previously described^37,47^. In brief, the proteins were activated by increasing the trap collision voltage from 50 to 240 V by 5 V increments. To compensate for slow traveling times due to reduced charge, IM-MS acquisition parameters were set as follows- wave velocity of 250 m/s, variable wave height between 0 to 30 V with a ramp time of 10%. IM-MS calibration for conversion of drift times to collision cross section values was done as previously described^46^ using the instrument parameters presented above.

### Theoretical Cross-Section Calculations

Theoretical collision cross section was calculated using the CCScalc function of Driftscope 2.5 (Waters).

### Data availability statement

Crystal structure of vinculin T12-A974K will be uploaded to the RCSB database and made available to everyone prior to publication. Attached to the submission are the PDB structure file, PDB validation report and structure statistics table.

## Electronic supplementary material

Supporting information
