# Supplementary material for: Conformational states during vinculin unlocking differentially regulate focal adhesion properties
Source: Sci Rep. 2018 Feb 9;8:2693. doi: 10.1038/s41598-018-21006-8 (PMC5807537; doi:10.1038/s41598-018-21006-8)
Supplement: Supplementary file 1 — Supporting information [file 41598_2018_21006_MOESM1_ESM.pdf]

Supplementary figures and tables

Conformational states during vinculin unlocking differentially regulate focal adhesion properties

Dror S. Chorev[1,2], Tova Volberg[1], Ariel Livne[1], Miriam Eisenstein[3], Bruno Martins[4], Zvi Kam[1], Brigitte M. Jockusch[5], Ohad Medalia[4,6], Michal Sharon[2,7] and Benny Geiger[1,7]

[1]Departments of Molecular Cell Biology, [2]Biomolecular Sciences, and [3]Chemical Research Support, Weizmann Institute of Science, Rehovot 76100, Israel, [4]Department of Biochemistry, University of Zurich, Winterthurerstrasse 190, Zurich 8057, Switzerland, [5]Department of Cell Biology, Zoological Institute, Technical University of Braunschweig, D-38092 Braunschweig, Germany and [6]Department of Life Sciences and the National Institute for Biotechnology in the Negev, Ben-Gurion University, Beer-Sheva 84105, Israel.

[7]Correspondening authors:

Benjamin Geiger	Michal Sharon
benny.geiger@weizmann.ac.il	michal.sharon@weizmann.ac.il
Tel: +972-8-934-3910	Tel: +972-8-934-3947

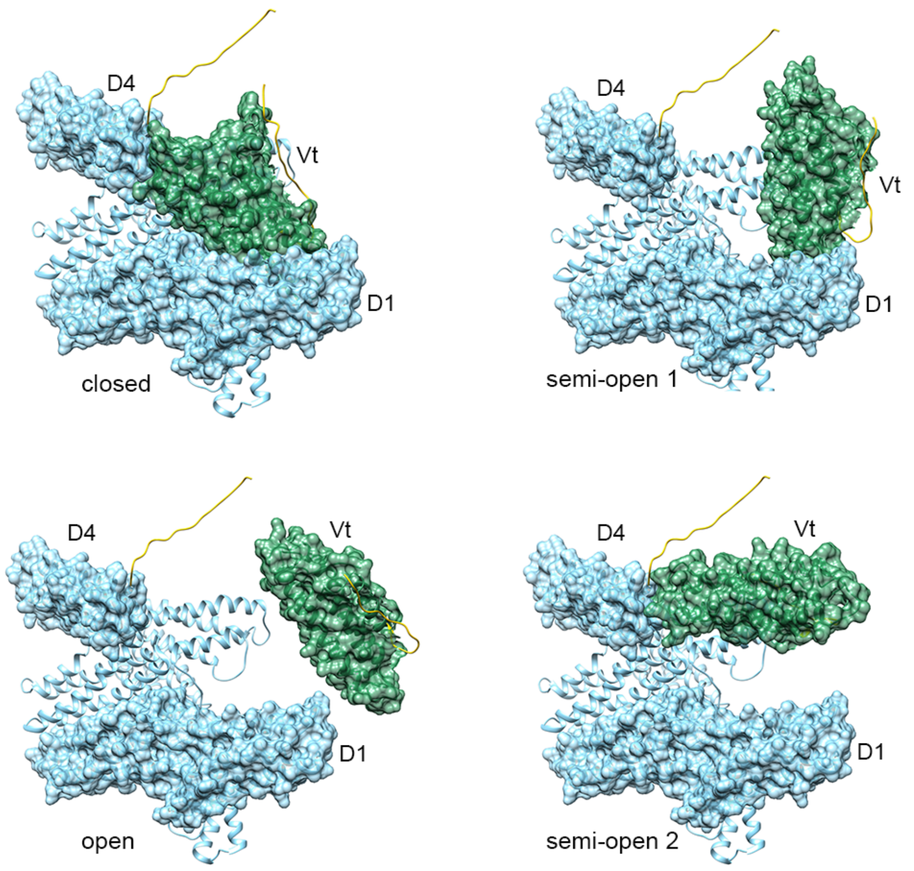

Supplementary Figure 1. Structural models of vinculin illustrating the stepwise detachment of Vt from Vh. The crystal structure of vinculin in the closed conformation, with Vt contacting both D1 and D4, corresponds to the C state (see Fig. 4). The calculated CCS value for this structure is 6490 Å^2. Two semi-open models are shown, one with Vt detached from D4 (semi-open 1) and another with Vt detached from D1 (semi-open 2); the calculated CCS for either of these models (7000Å^2 for semi-open 1 and 6968 Å^2 for semi-open 2) matched the experimental CCS value obtained for the SO state. However, based on the properties of the Vt-D1 and Vt-D4 interfaces, it is likely that the state SO corresponds to the semi-open 1 model. The CCS calculated for the open model (7362 Å^2) , with Vt detached from Vh, matches the observed CCS for state O; increasing the distance between Vt and Vh led to larger CCS values (up to 8325 Å^2, see text).

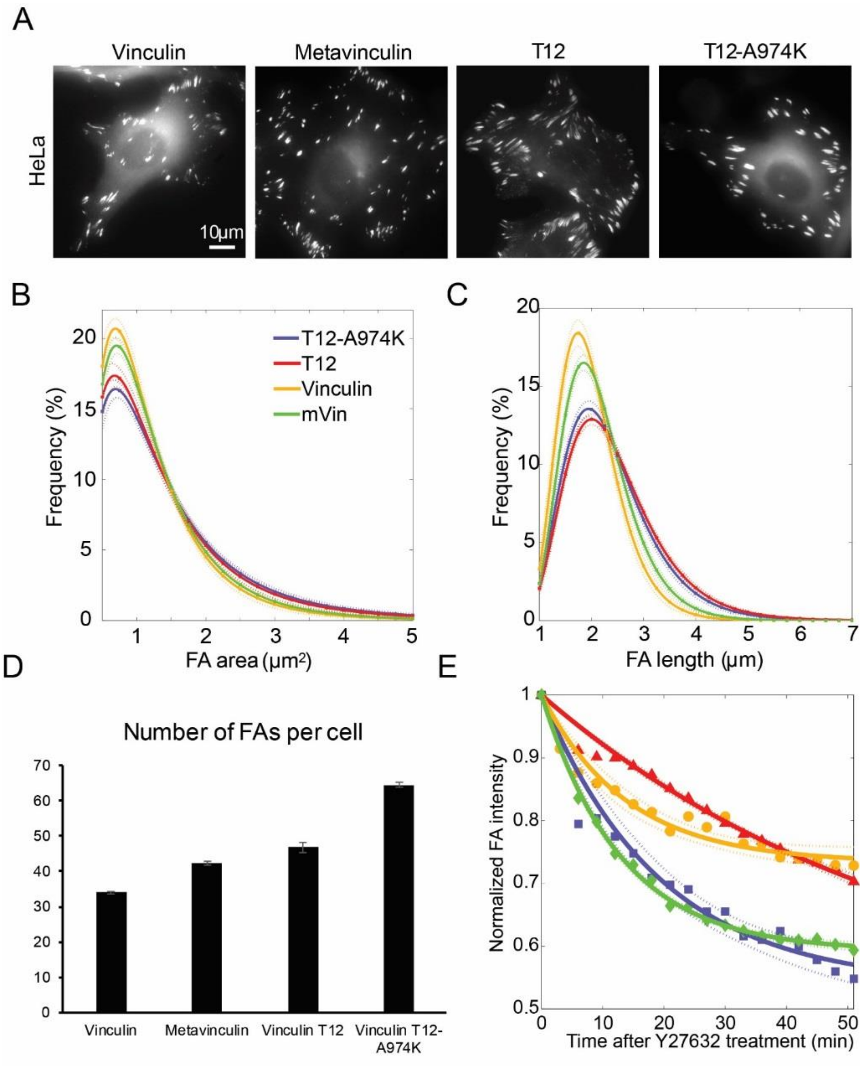

Supplementary Figure 2. Vinculin variants have distinct effects on FA morphology in HeLa cells. (A) The fluorescently-tagged vinculin variants localize to FAs and present different morphological attributes. Scale-bar is 10 µm. (B-D) Quantification of focal adhesion properties in HeLa cells, expressing the four vinculin variants. Solid curves represent best fits to log-normal distribution profiles with 95% confidence bounds (dashed lines). Error bars are standard deviation. (E) Time response of the different vinculin variants to Y27632 treatment in HeLa cells. Whereas T12 partially stabilized FAs, mVin and T12-A974K had the opposite effect of increasing FA instability. Solid curves are best fits to an exponential decay profile with 95% confidence bounds (dashed lines). Mean focal adhesion intensity was normalized with respect to its value just upon addition of 10µm Y27632 at time=0, and corrected for bleaching.

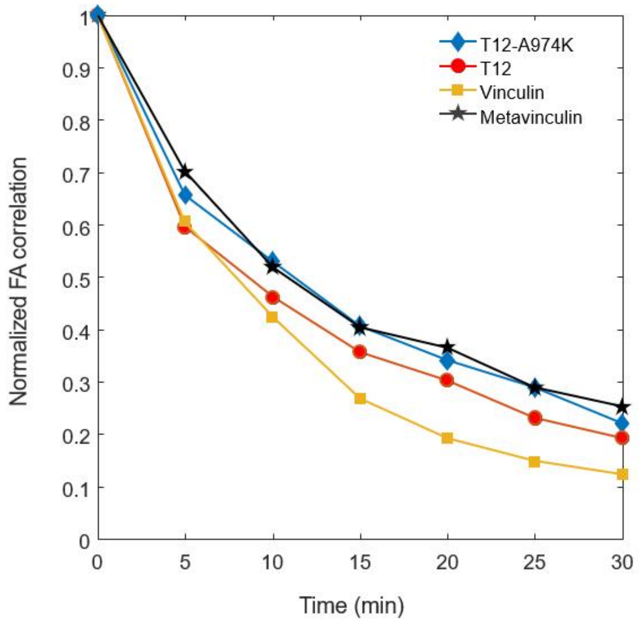

Supplementary Figure 3. Focal adhesion dynamics in vin -/- cells, transfected with the four vinculin variants. Time lapse movies (5 minutes intervals) of vin -/- cells, overexpressing each of the GFP conjugated vinculin proteoforms were segmented to create masks of FAs. FA dynamics was then quantified by comparing the different masks in each movie to the first one at time = 0. The correlation curves plotted here are the average of n>20 cells for each proteoform, with the correlation defined as the sum of the product of two masks, normalized by the sum of the first mask.

Table S1. Data collection and refinement statistics for vinculin T12-A974K variant.

	**T12-A974K**
**Wavelength (Å)**	1.0
**Resolution range**	19.91 - 3.0 (3.107 - 3.0)
**Space group**	P 31 2 1
**Unit cell dimensions (Å / °)**	97.81 97.81 233.77<br>90 90 120
**Completeness (%)**	97.89 (99.96)
**Redundancy (%)**	19.85 (18.45)
**Mean I/sigma(I)**	24.68 (1.61)
**Wilson B-factor**	97.94
**R-merge**	9.6 (206.3)
**CC1/2**	100 (69.5)
**Reflections used in refinement**	26203 (2603)
**Reflections used for R-free**	1311 (131)
**R-work**	0.2688 (0.3694)
**R-free**	0.3052 (0.4012)
**Number of non-hydrogen atoms**	7374
macromolecules	7368
ligands	1
solvent	5
**Protein residues**	965
**RMS (bonds)**	0.003
**RMS (angles)**	0.58
**Ramachandran favoured (%)**	94.96
**Ramachandran allowed (%)**	4.62
**Ramachandran outliers (%)**	0.42
**Clashscore**	9
**Average B-factor**	120.95
macromolecules	120.97
ligands	144.72
solvent	82.45
**Number of TLS groups**	4

Statistics for the highest-resolution shell are shown in parentheses.
